# Interleukin (IL)-4 Induces Leukocyte Infiltration In Vivo by an Indirect Mechanism

**DOI:** 10.1155/2009/193970

**Published:** 2009-09-10

**Authors:** Claude Ratthé, Jamila Ennaciri, David M. Garcês Gonçalves, Sonia Chiasson, Denis Girard

**Affiliations:** INRS-Institut Armand-Frappier, Université du Québec, Laval, Québec, Canada H7V 1B7

## Abstract

Interleukin (IL)-4 is a cytokine known mainly for its anti-inflammatory activity. Using the in vivo murine air pouch model, we found that IL-4 significantly increased the number of leukocytes after 9 hours of treatment, consisting mainly of neutrophil (60%) and monocytic (40%) cell populations. Using an antibody array, we found that the expression of several analytes (predominantly CCL2) was increased by IL-4 before the arrival of leukocytes. The IL-4-induced expression of CCL-2 was confirmed by ELISA. Air pouch resident lining cells were harvested and were found to express IL-4R*α*. CCL2 mRNA expression was monitored in lining cells, cells isolated from the air pouch skin, in RAW264.7 macrophage and in epithelial Mode-K cells and its expression was increased in response to IL-4 in all conditions. We conclude that IL-4 can attract leukocytes in vivo by an indirect mechanism involving the production of several analytes by, at least, resident cells.

## 1. Introduction

Neutrophils are the first blood leukocytes to arrive to an inflammatory site. The process of transendothelial migration from the blood to the tissue is complex and involves response to chemotactic factors and binding to adhesion molecules at the endothelium surface [1]. These events permit the extravasation of blood neutrophils to the tissue where they can perform different functions. Major roles of neutrophils include phagocytosis, production of reactive oxygen species, and production of chemoattractants such as IL-8, which recruit further competent cells [2]. Neutrophil response usually terminates when neutrophils undergo apoptosis and are phagocytosed by arising macrophages [3]. However, in certain inflammatory diseases, such as rheumatoid arthritis or inflammatory bowel diseases, neutrophils persist at the inflammatory site, and perpetuating the inflammation [4, 5]. 

 Interleukin-4 (IL-4) is a member of the CD132-dependent cytokines which also comprises IL-2, -7, -9, -15 and -21 [6]. There are two distinct IL-4 receptors: type I, which is composed of IL-4R*α* and CD132 (IL-2R*γ* or *γ*c), and type II receptor, composed of IL-4R*α* and IL-13R*α*1 chains [7]. IL-4 has largely been associated with anti-inflammatory functions since it decreases monocyte and macrophage functions [8, 9] and induces a Th_2_ type response. However, IL-4 can also be considered as proinflammatory, since its expression and a Th_2_ type response are associated with allergies and asthma [10], but it is still regarded mainly as an anti-inflammatory cytokine. IL-4 was also found to possess other proinflammatory functions, such as the recruitment of inflammatory cells by increasing the expression of VCAM-1 on the endothelial surface [11] and increasing eosinophils, macrophages and B cells chemoattraction [12–14]. This dual role can be of great importance for ameliorating or developing alternative therapies. For instance, IL-4 has been considered as a candidate for the treatment of rheumatoid arthritis, since it induces a Th_2_ biased response [15]. Paradoxically, other studies showed that IL-4 is implicated in the development of inflammatory arthritis [16], demonstrating the need for a better understanding of IL-4 proinflammatory functions. 

 Neutrophils are known to express the type I IL-4R (IL-4R*α*/CD132) but not the type II IL-4R (IL-4R*α*/IL-13R*α*1) [17–19]. Interestingly, IL-4 may possess both pro- and anti-inflammatory roles in neutrophils. For example, IL-4 induces production of an IL-1R antagonist considered to be an anti-inflammatory cytokine that blocks IL-1 effects [20]. The proinflammatory activity of IL-4 includes its ability to delay neutrophil apoptosis and the fact that it increases neutrophil IL-8 production [17], contributing to the recruitment of other leukocytes to site of inflammation. Studies have shown that the presence of neutrophils in asthma conjugated with increased IL-4 concentrations contribute to the severity of the disease [21, 22]. Even if many aspects of IL-4 effects on neutrophils have been studied, the ability to attract these cells in vivo is not well understood. In this study, we report that IL-4 possesses proinflammatory activities as judged by its ability to attract leukocytes in vivo and the local production of several proinflammatory molecules. 

## 2. Materials and Methods

### 2.1. Chemicals and Agonists

Recombinant murine IL-4 was purchased from R&D systems Inc (Minneapolis, MN). Lipopolysaccharide (LPS; from *Escherichia coli* 0111.B4) was purchased from Sigma Chemical Co. (St-Louis, MO), and Isoflurane was purchased from Pharmaceuticals Partners of Canada Inc (Richmond Hill, Ontario).

### 2.2. Neutrophils Isolation

Neutrophils were isolated from the venous blood of healthy volunteers by dextran sedimentation followed by centrifugation over Ficoll-Hypaque (GE Healthcare, Baie d’Urfé, Quebec, Canada) as previously described [19, 23, 24]. Blood donations were obtained from informed and consenting individuals according to institutionally approved procedures. Cell viability was monitored by trypan blue exclusion, and the purity (>98%) was verified by cytology from cytocentrifuged preparations coloured by Hema-stain.

### 2.3. Murine Air Pouch Model

C57BL/6 mice, 6 to 8 weeks of age, were obtained from Charles River Laboratories (St-Constant, Canada). On days 0 and 3, mice were anaesthetized with isofurane and 3 ml of sterile air was injected subcutaneously, in the back, with a 26-gauge needle to form an air pouch as published previously [25, 26]. On day 6, 1 ml of the following was injected into the air pouch: sterile HBSS, the positive control LPS (1 *μ*g/ml) or murine IL-4 (250 ng/ml), an optimal concentration determined previously when testing the effect of IL-4 in vitro [19]. In addition, this concentration was selected for comparison with those previously observed with IL-15 using the same in vivo model [25]. Mice were killed by CO_2_ asphyxiation 3, 6, 9, 12 or 24 hours after the treatment and the pouches were washed once with 1 ml and then twice with 2 ml of HBSS containing 10 mM EDTA. Exudates were centrifuged at 100 × g for 10 minutes at room temperature and supernatants were collected and stored at −80°C for further analysis. Cells were resuspended at 0.5 × 10^6^ cells/ml, centrifuged, spread onto microscope slides and stained with Hema-Stain for quantification of leukocyte subpopulations. To further characterize the leukocyte subpopulations, the cells were suspended in PBS containing 5 *μ*g/ml human IgG for 30 minutes at 4°C to block FcRs and then stained for 30 minutes at 4°C with purified rat antimouse 7/4 mAb directed against murine neutrophils or rat antimouse F4/80 Ag Ab directed against murine monocytes macrophages. Experiments were performed under protocols approved by Animal Use and Care Committees at INRS-Institut Armand-Frappier.

### 2.4. Cytokine/Chemokine Protein Array

Mouse inflammation antibody array I kit was purchased from Chemicon International (Temecula, CA) and all the steps for the simultaneous detection of 40 analytes were performed as per the manufacturer's recommendation. Previous experiments conducted with two membranes probed with the exudates from two different mice treated with LPS reveal that the results were reproducible (*data not shown*). Because of that, we next decide to pool exudates (*n* ≥ 13 mice) harvested from HBSS-treated (control) or IL-4induced murine air pouches to probe the membranes no more than two weeks after the in vivo experiments. The chemiluminescent signal from the bound cytokines/chemokines present in the exudates was detected on Kodak film X OMAT-RA. The signal intensity (each analytes in duplicate) of each spot was normalized to the membrane's positive control (four spots). Results are expressed as ratios (tested group/control). Ratios ≥1.2 were considered slightly positive and those ≥1.5 were considered strongly positive. Protein array membranes were scanned and densitometry analysis was performed using the Multi-Analyst program (Bio-Rad, Hercules, CA).

### 2.5. Detection of Murine CCL2 by ELISA

Fluids were harvested from air pouches after 6, 9, 12 and 24 hours of treatment with buffer or IL-4 and were pooled, correspondingly. CCL2 was quantified using the commercially available enzyme-linked immunosorbent assay (ELISA) kits for murine CCL2 (R&D systems Inc, sensitivity of <2 pg/ml) according to the manufacturer's recommendations. Samples were tested at least in duplicate.

### 2.6. Air Pouch Resident Cells

Air pouches were created on the back of C57BL/6 mice as previously described and cells lining the air pouch were harvested and stimulated using two different methods. First, 1 ml of trypsin-EDTA was injected in the pouches. After 5 minutes, cells were collected (hereafter referred to as lining cells) and the pouches were washed twice with 2 ml of HBSS-EDTA. Cells were centrifuged and incubated (10 × 10^6^ cells/ml) with HBSS or 250 ng/ml IL-4 for 6 hours. Cells were harvested and lysis and RNA extraction were performed as previously described. Second, 1 ml of HBSS or IL-4 was injected in the pouches. After 6 hours, the skin of the pouch was cut and the first layer of cells lining cells was manually removed and snap-frozen in liquid nitrogen (hereafter referred as skin cells). Tissues were mechanically crushed in liquid nitrogen using a pestle and mortar. Lysis and RNA extraction were performed as described [19].

### 2.7. Reverse Transcription and Polymerase Chain Reaction (PCR)

Cells (10 × 10^6^ cells/ml in RPMI-Hepes-P/S containing 10% fetal calf serum) were stimulated or not for 6 hours at 37°C with HBSS, or 250 ng/ml IL-4. Cells were harvested, washed three times with HBSS and RNA extraction was performed using the Absolutely RNA Miniprep Kit (Stratagene, La Jolla, CA) according to the manufacturer's protocol. The reverse transcription reaction was performed using 0.5 *μ*g of RNA and 200 U of the M-MLV enzyme in the following PCR buffer reaction: 5 mM MgCl_2_, 1 mM dNTP (dATP. dCTP, dGTP, dTTP), 40 U Rnase inhibitor, 0.03 U hexamer. PCR reactions were performed using the newly made cDNA, 0.5 U Taq Polymerase and the following primers: upstream: 5′-tccatgcaggtccctgtcatg-ctt-3′, downstream: 5′-ctagttcactgtcacactggtc-3′ for CCL2 (MCP-1); upstream: 5′-ctggcacctggagtgagtgg-3′, downstream 5′-acagcgcaccacact- gacact-3′ for IL-4R (IL-4R*α*); upstream: 5′-agagcaagcaccatgttgaaacta-3′, downstream tgggatcacaagattctgtaggtt for IL-2RG (CD132); upstream: 5′-tccgataacgaacgagactc-3′, downstream: 5′-cagggacttaatcaacgcaa-3′ for the 18 S control. Samples were loaded onto a 1.5% agarose gel containing 0.006% ethidium bromide. The bands were visualized using the Biorad Bioscan with a UV lamp and the results were analyzed using the Multi-Analyst program.

### 2.8. Cell Culture

Raw 264.7 and Mode-K cells were cultured in RPMI-1640 medium supplemented with 10% fetal calf serum, 2.5% 1M HEPES, 50U/ml penicillin, and 50 *μ*g/ml streptomycin. Cells were grown at 37°C, in a 5% CO_2_ incubator at concentrations ranging from 0.2 × 10^6^ cells/ml to 1 × 10^6^ cells/ml. Cells were grown to confluence in 6 well microplates and stimulated with HBSS or IL-4 for 6 hours at 37°C. Cells were scraped and washed twice with HBSS before performing lysis and RNA extraction as previously described [19].

### 2.9. Statistical Analysis

Statistical analysis was performed with SigmaStat for Windows Version 3.00 (Copyright 1992–2003 SPSS Inc.) using a one-way analysis of variance (ANOVA). Statistical significance was established at *P* < .05.

## 3. Results

### 3.1. ProInflammatory Effects of IL-4 In Vivo

**Figure 1:**
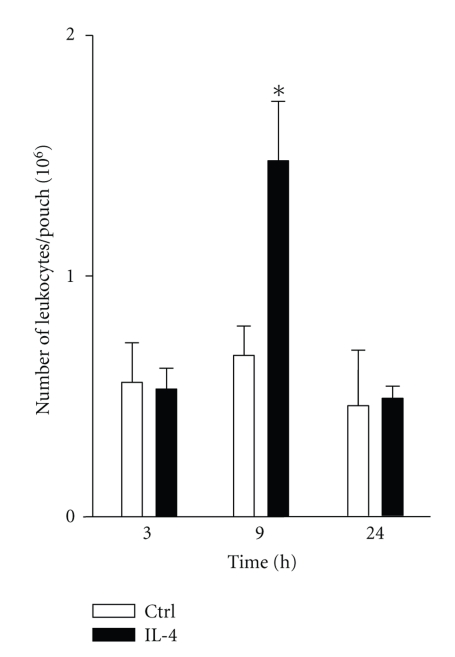
IL-4 induces inflammation in vivo*. *Murine air pouches were raised before injection of buffer (Ctrl) or 250 ng/ml IL-4 and exudates were harvested at the indicated periods of time and the number of leukocytes was calculated. Results are means ± SEM (*n* ≥ 6). *P < .05 versus Ctrl.

Previous studies demonstrated that IL-4 induces chemoattraction of different cell types such as eosinophils, macrophages, fibroblasts and B cells [27]. Here, we decided to investigate the ability of IL-4 to induce migration of neutrophils in vivo using the murine air pouch model. In this model, LPS is known to induce a potent acute inflammation peaking between 6–9 hours, consisting mainly in the recruitment of neutrophils (80–100%) disappearing (by a mechanism that remains unclear) after 12–24 hours [26]. Recently, using this model, we have identified that two other CD132-dependent cytokines, IL-15 and IL-21, can attract leukocytes in this model after 6–9 hours [25, 26], but unlike LPS, the response was less potent and these cytokines attract two major cell population: neutrophils (~60%) and monocytic cells (~40%). Therefore, we performed kinetic experiments from 3 hours to 24 hours. As shown in Figure 1, after 3 hours, the basal level of total leukocytes observed in control mice receiving the buffer was 0.56 ± 0.07 × 10^6^ cells/pouch (mean ± SEM) and was not increased by IL-4, eliminating the possibility that a rapid leukocyte influx occurs at earlier time points. However, after 9 hours of treatment, the number of leukocytes attracted by IL-4 was significantly increased when compared to control (1.5 ± 0.07 × 10^6^ for IL-4 versus 0.67 ± 0.03 × 10^6^ cells/pouch for control). Thus IL-4 induces migration of leukocytes in vivo. As expected, the leukocyte infiltration induced by IL-4 disappears overtime, and the number of attracted leukocytes returned to the basal level after 24 hours. 

**Figure 2:**
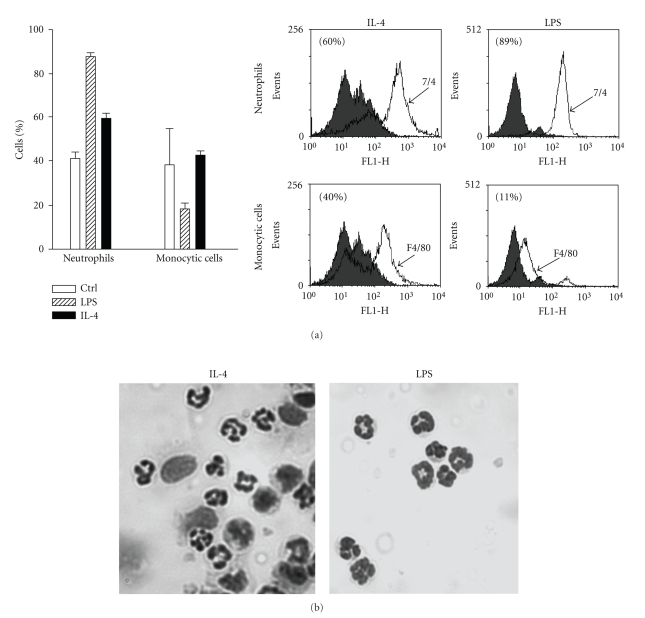
IL-4 induces the recruitment of neutrophil and monocytic cells in vivo. Murine air pouches were created and buffer, IL-4 (250 ng/ml) or LPS (1 *μ*g/ml) was administered for 9 hours as described in Materials and Methods. (a) cell populations were identified by flow cytometry (*n* ≥ 6) after staining with purified rat antimouse 7/4 mAb directed against murine neutrophils (neutro, open curve) or rat antimouse F4/80 Ag Ab recognizing murine monocyte/macrophages (mono, open curve) as described in Materials and Methods. Inset, representative data plotted in the bar graph, where the grey curves illustrate the appropriate isotypic controls. (b) cell identification was confirmed by cytology. Results are from one representative experiment out of at least 6.

 Because the two main leukocyte populations present in the exudates of the acute inflammation induced in the murine air pouch model by two other CD132-dependent cytokines, IL-15 and IL-21, are neutrophil and monocytic populations, we performed flow cytometry experiments with specific antibodies recognizing these two subpopulations. As shown in Figure 2(a), about 60% of cells attracted by IL-4 are neutrophils and 40% are monocytes macrophages. These results were confirmed by cytology (Figure 2(b)). As expected, more than 80% of cells attracted by LPS were neutrophils.

### 3.2. IL-4 Induces the Local Production of Several Analytes (Cytokines/Chemokines) In Vivo; Predominance of CCL2

Because of the previous results we performed an antibody array in order to monitor simultaneously 40 different analytes associated with inflammation in kinetic experiments. However, because we treated the mice with IL-4, and since this cytokine is among the tested analytes, we deliberately eliminated the corresponding data. Therefore, the total number of analytes is 39. As illustrated in Table 1, the levels of cytokine and chemokine expression detected after 3 hours of treatment with IL-4 were equivalent or even inferior to those obtained from control animals. However, after 6 hours of treatment with IL-4 the ratios of 14 of 39 analytes were above the level of control set to 1. Among these, 4 of 14 (CCL2/MCP-1, CXCL5/LIX, MIG and sTNFRI) exhibited a ratio ≥ than 1.2 (arbitrary selected). CCL2 is the most predominant chemokine detected in the exudates of IL-4-induced air pouches with a ratio of 7.25, followed by CXCL5, MIG and sTNFRI, with a ratio of 1.65, 1.46 and 1.46, respectively. After 9 hours of treatment, 23 of 39 analytes presented a ratio greater than controls. Among these, 17 of 22 analytes presented a ratio ≥ 1.2. In contrast to the 6 hours time point, there were no analytes detected with a ratio greater than 4. In fact, it appears rather that there are more analytes present in the exudates with a ratio ranging from 1.2–2.0. IL-12p70 and IL-13 are the two cytokines detected in greater concentrations with ratios of 2.1 and 2.0, respectively. After 12 hours, only 5 of 39 analytes exhibited a ratio above 1 and only three, KC, leptin and MIP-1*α*, had a ratio greater than 1.2, each at 1.44. 

### 3.3. IL-4 Increases CCL2 Protein Expression In Vivo

**Figure 3:**
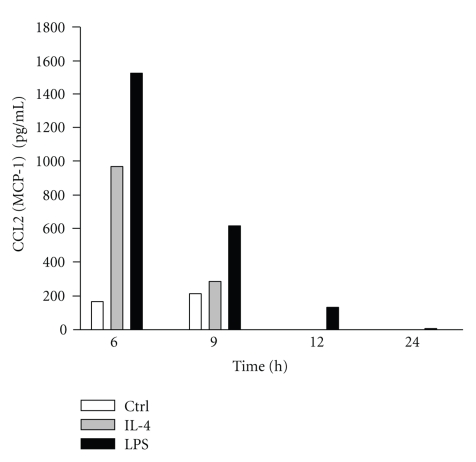
IL-4 increases CCL2 protein expression in the exudates. Air pouches was created as described in Materials and Methods and 1 ml of the buffer (Ctrl), LPS (1 *μ*g/ml), or IL-4 (250 ng/ml) were administered into the pouch. Exudates were harvested after 6, 9, 12 and 24 hours, pooled (*n* ≥ 7) and then CCL2 production was measured by ELISA.

Because of the above results, we next focused on CCL-2. In order to confirm the results obtained by the antibody array assay, the exudates were tested by a specific ELISA for murine CCL-2. As illustrated in Figure 3, the concentration of CCL2 detected in the pooled air pouch fluids (*n* ≥ 7 mice) was increased by IL-4 when compared to controls. Interestingly, at the 6 hours and 9 hours time points, the concentration of CCL2 was 975 (IL-4) versus 167 (control) pg/ml for a ratio of 5.8 and 288 (IL-4) versus 214 pg/ml for a ratio of 1.4, respectively. These values are close to the ratios obtained by the antibody array assay (7.25 and 1.54, resp.). Of note, CCL2 was not detected after 12 hours. LPS was used as a positive control.

### 3.4. Expression of IL-4R in Murine Air Pouch Lining Cells and Production of Cytokines in Response to IL-4

**Figure 4:**
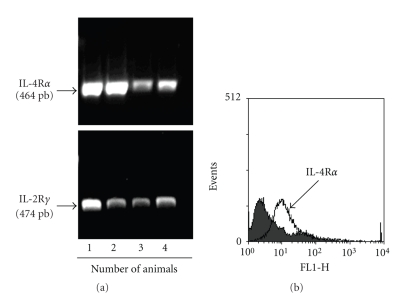
The murine air pouch resident lining cells express the IL-4 receptor*. *Air pouches were created and lining cells were harvested as described in Materials and Methods. IL-4R*α* chain was detected by RT-PCR (a) and by flow cytometry (b) using a specific antibody directed against murine IL-4R*α*. Note that IL-4R*α* mRNA was detected at variable levels in all tested animals, as well as IL-*2*R*γ*. Only four different animals are presented (nos. 1–4). The receptor was detected on the cell surface of the lining cells. Results are from one representative experiment out of 4.

**Figure 5:**
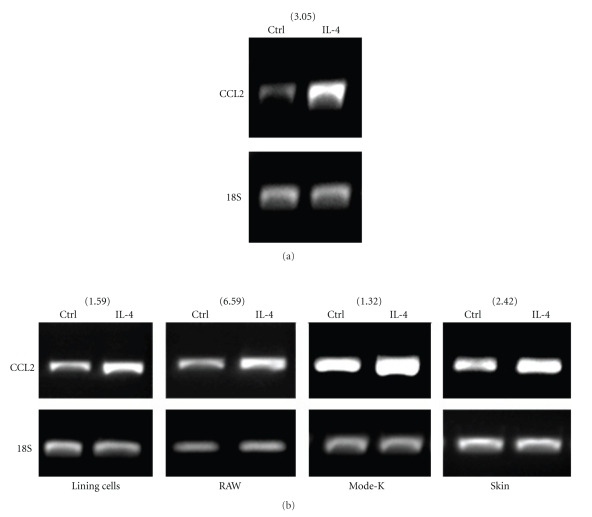
*Expression of CCL2 mRNA in different cell populations. *(a) air pouches were created and IL-4 or buffer was administered for 6 hours. Cells were harvested, pooled (*n* = 6) and CCL2 (MCP-1) mRNA were detected as described in Materials and Methods. (b) Murine air pouches were created and the lining cells and skin cells were harvested as described in Materials and Methods. Lining cells, skin cells, murine macrophages (RAW) and epithelial Mode-K cells were stimulated for 6 hours with buffer (Ctrl) or 250 ng/ml IL-4 and the expression of CCL2 mRNA was performed by RT-PCR. Results are from one representative experiment out of at least 3. Number between parentheses represents the ratio of the signal intensity of (IL-4/18S)/Ctrl/18S) obtained after densitometry analysis using a Fluor-S multi-imager (Bio-Rad, Hercules, CA) and the Multi-Analyst version 1.1 program.

Because several analytes detected in the exudates of IL-4-induced air pouch were present before the emigration of leukocytes, and since most of them are not necessarily typical neutrophil or monocyte chemoattractants, it is highly plausible that the lining or resident cells from the pouch are the first to interact with IL-4. Therefore, we isolated the lining cells from air pouches and investigated the expression of IL-4R. As illustrated in Figure 4 the lining cells expressed both CD132 (IL-2R*γ*) and IL-4R*α*, as assessed by RT-PCR (Figure 4(a)). We confirmed the expression of IL-4R*α* at the cell surface of lining cells by flow cytometry (Figure 4(b)). Of note, expression of this component alone is sufficient to induce a signal in response to IL-4. Knowing that the harvested lining cells express the IL-4R, we next stimulated them in vitro (pool of *n* = 9) with IL-4 for 6 hours and investigated the expression of the same analytes. As illustrated in Table 2, 20 of 39 analytes were detected with a ratio greater than 1. Among these, 5 (Eotaxin-2, G-CSF, MIG, SDF-1 and sTNFR-I), exhibited a ratio ≥ 1.2. Of note, CCL2 was only detected at a ratio of 1.12. This suggests that the high level expression of several analytes observed in vivo in IL-4-induced air pouch necessitates other factors and/or players. Thus, the profile of analytes detected after 6 hours in response to IL-4 differs in terms of expression level and in terms of specificity in vivo and in vitro, suggesting that other cells are involved and that the responses are context specific. Because CCL2 is the predominant analyte detected in the exudates in response to IL-4, and in order to better elucidate the participation of different cell populations for such production, we first verified the possibility that the attracted leukocytes themselves participate in the increased production of CCL2. As illustrated in Figure 5(a), leukocytes attracted by IL-4 can produce higher levels of CCL2 mRNA when compared to controls. We next collected the lining cells and the skin cells from the inside part of the air pouch treated with buffer or IL-4 and monitored the gene expression of CCL2. In addition, we investigated the gene expression of CCL2 in murine RAW 264.7 macrophages and epithelial Mode-K cells. As illustrated in Figure 5(b), IL-4 increased the CCL2 mRNA expression in all conditions when compared to controls. Thus, according to the results obtained for CCL2, the production of the different analytes induced by IL-4 could originate from resident cells expressing IL-4R, as well as from leukocytes attracted by IL-4.

## 4. Discussion

Several members of the CD132-dependent cytokines, including IL-2, IL-4, IL-7, IL-9, IL-15 and IL-21, are involved in diverse inflammatory disorders [18]. Among these, IL-4 is best known for its anti-inflammatory activity. For example, IL-4 was found to induce production of interleukin-1 receptor antagonist (IL-1Ra) in neutrophils [28] and to antagonize the biological action of IL-1 by inducing the expression and release of IL-1 type II decoy receptor [29]. In addition, IL-4 was found to inhibit LPS-induced prostaglandin E_2_(PGE_2_) as well as LPS-induced cyclooxygenase-2 (COX-2) protein expression in neutrophils [30]. Consistent with its anti-inflammatory properties, Wertheim and colleagues [31] reported that IL-4 inhibited IL-8 production, but this was observed in LPS-primed neutrophils. In contrast, we have demonstrated that IL-4 can directly increase IL-8 production in human neutrophils [17]. The identification of IL-4 as a neutrophil agonist is not recent. For example, more than 18 years ago, Boey and colleagues [32] reported that IL-4 enhanced neutrophil-mediated killing of opsonized bacteria. It is clear that the biological activity of IL-4 in neutrophils reported in the literature is far from complete. In addition, its proinflammatory activity remains to be better elucidated. 

 Using the murine air pouch model, we demonstrate that IL-4 possesses proinflammatory activity in vivo based on its ability to attract leukocytes, including neutrophils. It is important to mention that very few studies have investigated the ability of IL-4 itself to promote an inflammatory response in vivo. However, in one study, IL-4 was found to induce leukocyte recruitment in vivo using the cremasteric postcapillary venule model. Also, although IL-4 was found to attract eosinophils, monocytes and lymphocytes in this model, no neutrophils were attracted. Interestingly, other CD132-dependent cytokines, including IL-15 and IL-21, were previously found to attract leukocytes in vivo in this model [25, 26]. As for IL-4, IL-15 is known as a neutrophil agonist but, unlike these two cytokines, we recently documented that IL-21 does not modulate a panel of neutrophil functions and this was correlated with the absence of the IL-21R*α* subunit in these cells [26]. Also, even if IL-21 is not a direct neutrophil agonist, this cytokine was found to attract neutrophil and monocytic cell populations in vivo. While IL-15 was found to increase the local production of IL-6 and MIP-2 (CXCL2) into the cell-free air pouch exudates, IL-21 did not. Moreover, IL-21 did not increase CCL3 (MIP-1*α*) and CCL5 (RANTES) production. This suggests that different cytokines and chemokines are involved in the neutrophil recruitment and that resident cells, in the case of IL-21, play an important role. Here, using an antibody array assay, we found that administration of IL-4 into air pouch increased local production of several analytes. Interestingly, the production of CCL2, CXCL5, MIG and sTNFR I were detected in higher concentrations in IL-4-treated pouches before the total leukocyte counts were significantly increased versus controls. Thus, IL-4 cannot only directly attract neutrophils in vitro; it can also act indirectly in vivo. Results from Wan and colleagues are in agreement with this latter point [33]. They found that CCL2, a chemokine previously thought to attract leukocytes other than neutrophils, could enhance leukocyte rolling, adhesion and recruitment in vivo using intravital microscopy of the mouse cremaster microcirculation system where about 85% of cells attracted were neutrophils. As stated by the authors, endothelial cells were probably not involved, based on the inability of CCL2 to increase P-selectin expression in these cells. They propose an indirect effect involving activation of an intermediary tissue cell. The role of CXCL5 in neutrophil attraction is well established. CXCL5 is the murine equivalent of human ENA-78, a potent activator of neutrophil function known to possess neutrophil-activating properties similar to those of IL-8 [34]. MIG is a CXC chemokine (CXCL9) induced in different cells, including parenchymal cells, endothelial cells and macrophages, primarily in response to Interferon-*γ* (INF-*γ*) explaining why this chemokine was named MIG for monokine-induced by Interferon-*γ*. Curiously, in our present study, the IL-4-induced MIG production was transitory and not accompanied by a significantly increased expression of IFN-*γ* (Table 2). The IL-4-induced production of sTNFR I suggests that IL-4 may also induce anti-inflammatory signals in parallel. Interestingly, we have previously demonstrated that the proinflammatory cytokine IL-15 induced, in parallel, an anti-inflammatory response based on the production of IL-1Ra [35]. 

 Monocyte chemoattractant protein-1 (MCP-1/CCL2) is the analyte that has the highest expression after 6 hours of injection of IL-4 in the pouches. It has been demonstrated that CCL2 protein expression could be increased under certain conditions [36]. In contrast, production of CCL2 was also reported to be decreased under other circumstances [37]. CCL2 is known to be produced by endothelial cells [38] in response to IL-4 and these cells could be an important source of this chemokine in our experiments. CCL2 mostly attracts macrophages and monocytes [39]. 

 Interestingly, a distinct set of analytes was detected in the supernatants of IL-4-induced lining cells. However, the increased signals of some analytes, including IFN-*γ*, IL-3, I-TAC, leptin, CCL2, MIP-1*γ* and sTNFR I were common to both conditions (IL-4-induced air pouches and IL-4-induced lining cells) when compared with their respective controls. Of note, IL-4 was found to markedly increase the production of eotaxin-2, MIG and SDF-1 by these lining cells, indicating that production of these analytes could be under the control of a selective mechanism in vivo that is lost in vitro when these cells are isolated from their natural environment. This is a good example illustrating the complexity of the regulation of cytokines and chemokines in vivo in response to a unique cytokine like IL-4. 

 The results obtained regarding the expression of CCL2 mRNA in IL-4-induced Mode-K, RAW-264.7, lining and skin cells, demonstrate further the complexity of the response occurring after IL-4 treatment and support the role of cells of different origins for attracting neutrophil and monocytic cell populations in vivo. IL-4 was found to markedly increase CCL2 mRNA in all tested conditions. The decreased production of CCL2 observed after 9 hours (versus 6 hours) could be explained in part by the increased expression of IL-13 which is known to downregulate the MCP family member's production [37]. We also observed that some factors known to activate or recruit neutrophils are present in the extracts of pouches that were stimulated with IL-4. For example, there was an increased expression of keratinocyte cytokine (KC), a known neutrophil chemoattractant and activator, and macrophage inflammatory protein-1 alpha (MIP-1*α*), which induces superoxide production in neutrophils in vitro, and leptin, known to delay neutrophil apoptosis [40–42]. These three analytes are still detected in greater amounts 12 hours after IL-4 administration, but the ratio of the signal intensity is less than 1.5. 

 In conclusion, this study provides the first evidence that IL-4 promotes the recruitment of neutrophils and monocytic cells in vivo by an indirect mechanism. We propose that the IL-4R positive murine air pouch resident cells (including lining + skin cells), are the first cells to be activated by IL-4 and that these cells will induce the production of different analytes in the exudates, some of which will attract neutrophils that will participate, as do monocytes, in the local production of analytes leading to the attraction of other neutrophils and/or monocytes.

## Figures and Tables

**Table 1:** Analysis of the different analytes detected in the cell free exudates harvested from IL-4-induced air pouch^1^.

Analyte	3 hours	6 hours	9 hours	12 hours
BLC	0.23	1.10*	0.86	nd
CD30L	0.47	nd	1.71*	nd
Eotaxin	0.70	1.10*	1.29*	nd
Eotaxin-2	0.70	0.85	1.36*	0.72
Fas Ligand	0.23	0.55	0.43	nd
Fractalkine	0.16	0.55	1.00	nd
G-CSF	0.31	nd	1.20*	nd
GM-CSF	0.93	1.10*	0.43	0.36
IFN*γ*	0.47	1.10*	0.64	nd
IL-1*α*	nd	1.10*	0.96	0.72
IL-1*β*	nd	nd	0.86	nd
IL-2	nd	nd	0.86	nd
IL-3	nd	1.10*	1.71*	nd
IL-6	nd	nd	1.71*	nd
IL-9	nd	0.55	1.71*	nd
IL-10	nd	nd	1.71*	nd
IL-12 p40/p70	0.35	0.55	1.03*	nd
IL-12p70	0.23	nd	2.00*	0.72
IL-13	0.93	1.10*	2.14*	0.72
IL-17	0.47	0.55	0.86	nd
I-TAC	nd	1.10*	1.20*	0.72
KC	nd	0.55	1.71*	1.44*
Leptin	nd	1.10*	1.50*	1.44*
LIX/CXCL5	0.93	1.65*	0.95	0.72
Lymphotactin	nd	0.70	1.19*	0.72
MCP-1/CCL2	0.47	7.25*	1.54*	nd
M-CSF	nd	nd	**1.29**	0.72
MIG	nd	1.46*	0.94	nd
MIP-1*α*	0.47	0.37	1.71*	1.44*
MIP-1*γ*	0.58	1.17*	1.05*	1.15*
RANTES	0.78	0.55	1.16*	0.12
SDF-1	0.47	nd	0.86	0.36
TCA-3	0.70	0.55	0.86	0.72
TECK	nd	nd	0.86	nd
TIMP-1	0.23	0.78	1.04*	nd
TIMP-2	0.47	nd	1.29*	nd
TNF-*α*	nd	0.55	0.86	0.72
sTNFR I	0.45	1.46*	0.97	1.08*
sTNFR II	nd	0.55	0.86	nd

^1^Air pouches were created and 250 ng/ml IL-4 or buffer was administered into the pouch for the indicated periods of time. Pooled exudates (*n* ≥ 13 mice) were used to probe the membranes (Mouse inflammation antibody array I) no more than two weeks after the in vivo experiments. Results are calculated as ratios, as described in Materials and Methods. nd, not detectable; *, ratio above 1; numbers in bold characters, ratio ≥ 1.2.

**Table 2:** Analysis of the different analytes detected in the supernatant harvested from IL-4-induced lining cells^1^.

Analyte	6 hours
BLC	0.36
CD30L	0.54
Eotaxin	0.54
Eotaxin-2	2.92*
Fas Ligand	0.36
Fractalkine	1.08*
G-CSF	1.20*
GM-CSF	0.72
IFN*γ*	1.08*
IL-1*α*	0.98
IL-1*β*	1.08*
IL-2	1.08*
IL-3	1.08*
IL-6	0.85
IL-9	1.08*
IL-10	1.08*
IL-12 p40/p70	0.81
IL-12p70	0.81
IL-13	0.72
IL-17	0.54
I-TAC	1.08*
KC	1.14*
Leptin	1.08*
LIX/CXCL5	0.99
Lymphotactin	0.54
MCP-1/CCL2	1.12*
M-CSF	0.54
MIG	2.16*
MIP-1*α*	1.08*
MIP-1*γ*	1.03*
RANTES	0.52
SDF-1	2.16*
TCA-3	0.75
TECK	0.54
TIMP-1	0.65
TIMP-2	1.08*
TNF-*α*	0.54
sTNFR I	1.24*
sTNFR II	1.08*

^1^Air pouches were created and lining cells were harvested. pooled and incubated in vitro with 250 ng/ml IL-4 for 6 hours. The supernatant was used to probe the membranes (Mouse inflammation antibody array I) no more than two weeks after the in vivo experiments. Results are calculated as ratios, as described in Materials and Methods. *, ratio above 1; numbers in bold characters, ratio ≥ 1.2.
